# Comparison of Preservatives for the Prevention of Microbial Spoilage of Apple Pomace During Storage

**DOI:** 10.3390/foods14142438

**Published:** 2025-07-10

**Authors:** Ashley Harratt, Wenyuan Wu, Peyton Strube, Joseph Ceravolo, David Beattie, Tara Pukala, Marta Krasowska, Anton Blencowe

**Affiliations:** 1 Applied Chemistry and Translational Biomaterials (ACTB) Group, Centre for Pharmaceutical Innovation (CPI), UniSA Clinical and Health Sciences, University of South Australia, Adelaide, SA 5000, Australia; 2 Future Industries Institute (FII), UniSA Science Technology Engineering and Mathematics (STEM), University of South Australia, Mawson Lakes, SA 5098, Australia; 3 Ashton Valley Fresh, Lobethal Rd, Ashton, SA 5137, Australia; 4 School of Physics, Chemistry and Earth Sciences, University of Adelaide, Adelaide, SA 5005, Australia

**Keywords:** apple pomace, preservative, spoilage, fermentation, microbiology

## Abstract

Apple pomace, a by-product from the production of concentrated juice, is a major contributor to global food waste. Despite its beneficial nutritional profile, apple pomace is predominantly disposed of in landfills. Rapid fermentation and spoilage caused by microorganisms are compounding factors in this demise, despite significant research into upcycling strategies. Thus, there is an unmet need for economical approaches that allow for the preservation of pomace during storage and transportation to centralized processing facilities from regional hubs. To address this challenge, we investigated the potential of different preservatives for preventing microbial growth and the spoilage of apple pomace, including antimicrobials (natamycin and iodine), polysaccharides (chitosan and fucoidan), and acetic acid. Spread plates for total microbial and fungal counts were employed to assess the effectiveness of the treatments. High concentrations (10,000 ppm) of chitosan were effective at reducing the microbial load and inhibiting growth, and in combination with antimicrobials, eliminated all microbes below detectable levels. Nevertheless, acetic acid at an equivalent concentration to commercial vinegar displayed the highest economic potential. Apple pomace submerged in 0.8 M acetic acid (3 kg pomace per liter) resulted in a five-log reduction in the microbial colony-forming units (CFUs) out to 14 days and prevented fermentation and ethanol production. These results provide a foundation for the short-term storage and preservation of apple pomace that could contribute to its upcycling.

## 1. Introduction

In addition to being one of the most consumed fresh fruits worldwide, apples are also extensively used in the production of concentrated juice for beverages, fruit snacks, and sweets [1], resulting in the generation of an estimated 4 million tons/year of apple pomace [2]. Apple pomace, along with other fruit and vegetable waste and by-products, is recognized as a significant global problem, which, if dealt with responsibly through upcycling and valorization, could contribute to the long-term sustainability of the food supply chain [3,4]. Apple pomace contains a host of beneficial nutrients and bioactive compounds, including polyphenolic compounds with anti-oxidant and anti-inflammatory properties [5]. Regardless, the upcycling of apple pomace can be challenging due to spoilage, and the majority is disposed of in landfills, resulting in potential environmental contamination and soil degradation, or used as animal feed [2,6]. Nevertheless, significant research efforts have been directed toward the extraction of bioactive compounds, fermentation bioprocesses, and the integration of apple pomace in foods and cosmetics [1,5,7]. Despite the emerging applications, transporting apple pomace to processing centers at scale and storage is expensive and highly time sensitive due to microbial spoilage [8].

While significant amounts of apple pomace are generated regionally, there is utility to being able to transport and store pomace for centralized processing, either for upcycling in products for human consumption, the extraction of bioactives, or processing with other by-products to make nutritional animal feed. However, the high water content and high concentration of fermentable sugars in apple pomace, in combination with naturally abundant microorganisms, results in the rapid fermentation and spoilage of pomace over several days post-pressing [9]. Whilst this presents opportunities for bioethanol production, during storage and transport, this can be undesirable and result in the release of unpleasant odors, environmental pollutants, and greenhouse gas emissions [9,10].

To address this challenge, we were interested in investigating alternative approaches for the short-term preservation of pomace. A common approach to prevent spoilage and allow longer-term storage involves drying apple pomace; however, this is energy-intensive and costly [6,11]. Alternatively, the addition of preservatives may be a viable option to reduce the microbial load and prevent fermentation and spoilage. While there is a lack of research on this subject for apple pomace, a wide range of preservatives are often used for other fruit and vegetable products. These commonly include natural preservatives, such as salt, sugar, honey, vinegar, vegetable oil, and spices, and chemical preservatives, such as sulfites and benzoates [12]. Depending on the intended use of the apple pomace, some of these preservatives (e.g., salt, sugar, and oil) may be undesirable. Other natural preservatives that may be applicable include anti-fungal and anti-bacterial compounds such as natamycin and nisin, respectively [13], which are commonly used in dairy products, canned vegetables, and juices.

Recently, there has been growing interest in the application of polysaccharide coatings for the preservation of whole fruits and vegetables, with some inhibiting microbial growth and delaying aging [14,15]. In particular, the bactericidal and bacteriostatic activity of chitosan as a food preservative has been well studied [16], although the use of chitosan in solution has provided mixed results. Anacarso et al. studied the anti-listerial activity of chitosan solutions on artificially contaminated fruit and vegetables, which was highly effective on apples (>5-log reduction in the CFU per gram (CFU/g)), but less so on zucchini, corn, or radishes (1.5-log reduction CFU/g) [17]. Isolated from brown seaweed, fucoidan is another polysaccharide with potentially interesting anti-bacterial activity [18] and has been extensively studied as a bioactive ingredient for functional foods, with prebiotic, anti-oxidant, anti-inflammatory, and anti-viral activity, to mention a few [19]. Similarly to chitosan, fucoidan has been studied as an edible coating for fruits, demonstrating anti-bacterial activity and an extended shelf-life [20]. The antimicrobial activity of fucoidan has also been demonstrated in beverages, with concentrations of 100–1000 ppm showing a bactericidal effect in a pasteurized apple beverage [21].

In this work, we investigated several different preservatives for apple pomace with the aim of identifying a cheap and effective preservative that would inhibit microbial growth and delay spoilage, potentially allowing for the short-term storage and transportation of pomace prior to further processing or its use as animal feed. Specifically, we studied the ability of conventional preservatives (e.g., natamycin and acetic acid) to reduce the total microbial and fungal load and compared them to emerging additives such as chitosan and fucoidan, and a broad-spectrum antimicrobial agent, iodine. While iodine is not typically used as a preservative in food or beverages, it is an important micronutrient that is often added to fortify products. Whether or not iodine displayed antimicrobial activity in apple pomace at safe and regulatory levels was to be determined. To the best of our knowledge, this is the first study to investigate a broad range of preservatives with different mechanisms and preservation methods for apple pomace, and it provides important findings for the preservation of other food waste or by-products.

## 2. Results and Discussion

### 2.1. Microbial Load and Growth on Apple Pomace

Apple pomace was supplied by Ashton Valley Fresh, who process apples—predominantly Pink Lady, Granny Smith, Royal Gala, and Kanzi varieties—from the Adelaide Hills region, South Australia. To assess the effect of different preservatives on the endogenous microflora and spoilage of apple pomace during storage, microbiological plate counting was performed on plate count agar (PCA) and dichloran rose Bengal chloramphenicol (DRBC) agar to determine the total microbial and fungal (yeasts and molds) CFUs, respectively. Prior to introducing different treatments, initial experiments were conducted with fresh apple pomace pressed on four separate days to evaluate the batch-to-batch variability. The pomace was blended with water (1:3 w/w ratio) to form a paste, and each batch was analyzed immediately on the day it was collected (day 0) and following three days (day 3) of storage at ambient temperature (22 ± 1 °C) in a sealed container. Under these conditions, the apple pomace was found to have a pH of 3.4 on day 0 that decreased to ~3.0 on day 3 (Supplementary Information (SI), Figure S1), possibly indicating the presence of lactic acid bacteria. In addition to the PCA and DRBC plates, violet red bile agar (VRBA) plates were also used to assess for the presence of coliforms. On day 0, 1.4 × 10^5^ (5.0% relative standard deviation (RSD)) and 4.6 × 10^4^ (21% RSD) CFU/mL were present on the PCA and DRBC plates, respectively, which increased significantly to 7.5 × 10^7^ (3.4% RSD) and 7.0 × 10^7^ (9.8% RSD) CFU/mL on day 3, most likely due to the high water and sugar content. These results indicated the presence of both bacteria and fungi in the apple pomace, and that the microbial load between batches is relatively consistent. No growth was detected on the VRBA plates, indicating the absence of coliforms, and therefore, these plates were excluded from subsequent experiments. All experiments conducted with preservatives included untreated apple pomace as a control and baseline for microbial growth.

### 2.2. Endogenous Microflora in Apple Pomace from the Adelaide Hills Region

The microbiome associated with apples varies significantly depending on cultivar and geographical location [22], and even within the different tissues (i.e., stem, peel, fruit, seeds, and calyx) [23]. Therefore, matrix-assisted laser desorption ionization time-of-flight (MALDI ToF) mass spectrometry was initially employed to analyze the apple pomace and identify species of bacteria and fungi that were present. Several species of the Gram-negative *Pseudomonas* bacterial genera were identified, including *P. tolaasii*, *P. taetrolens*, *P. fragi*, and *P. fluorescens*. In addition, Gram-negative *Pantoea agglomerans* and *Rahnella aquatilis* were identified, which, along with *P. fluorescens*, are particularly favorable for biological control. *P. agglomerans* has been reported to control fire blight disease caused by *Erwinia amylovora* in apple trees [24], while *R. aquatilis* and *P. fluorescens* control post-harvest mold growth in apples caused by pathogenic *Penicillium* and *Botrytis* species [25,26]. Fungal species that were present in the apple pomace included the yeasts, *Hanseniaspora uvarum*, *Metschnikowia pulcherrima*, *Pichia kudriavzevii*, and *Saccharomyces cerevisiae*, all of which are commonly found on fruit and are often responsible for their fermentation and spoilage [10,27,28]. Interestingly, the intentional addition of *H. uvarum* and *S. cerevisiae* to apple pomace has been studied for bioethanol production and the formation of aroma compounds that could potentially be used as natural flavoring additives [10]. Microscopy of spread plates also identified the presence of fungal Penicillium and Fusarium genera, known for causing post-harvest mold and core rot in apples [29,30].

### 2.3. Treatment of Apple Pomace with Antimicrobials

**Figure 1:**
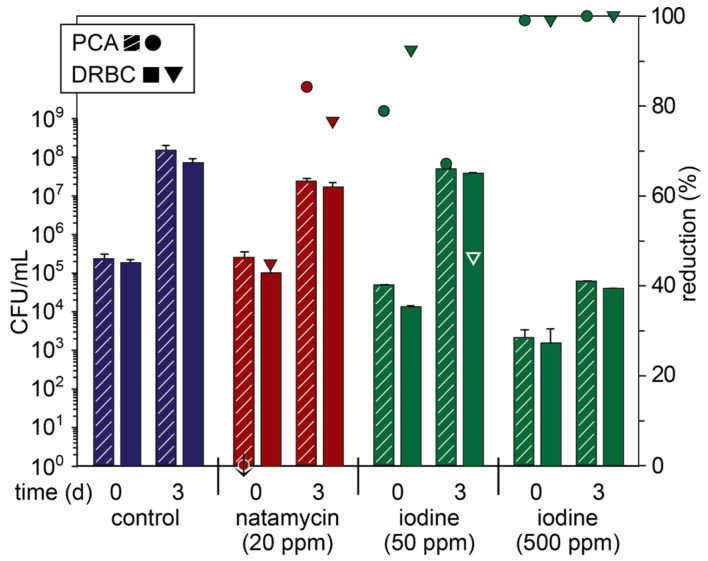
Microbial count (primary axis; bars) of fresh apple pomace (control) and pomace treated with natamycin and iodine plated on PCA and DRBC plates on day 0 (day of pressing) and day 3 (stored at ambient temperature) and percentage reduction in microbial count relative to the control on that day (secondary axis; symbols). Symbols marked with a ↓ indicate an increase in the microbial count relative to the control.

The blended apple pomace was firstly treated with natamycin (20 ppm) and iodine (50 ppm) in the form of potassium triiodide (KI_3_, Lugol’s solution). While natamycin is used extensively as a preservative in foods and beverages to prevent fungal growth [31], iodine is a broad-spectrum antimicrobial agent predominantly used to treat open wounds, although it is also important as a trace micronutrient for mammals [32]. These concentrations were selected as natamycin has been reported to be effective against yeasts and molds in fruit juices at 10–20 ppm [31], and the Food Standards Australia New Zealand recommends the fortification of salt with iodine concentrations in between 25 and 65 ppm [33]. Immediately following treatment on day 0, natamycin resulted in a small reduction (45%) in fungal counts, but there was little change in the overall microbial count, which is consistent with the activity of natamycin on yeasts and molds (Figure 1 and SI, Tables S1 and S2) [31]. Although there was a significant increase in the microbial load on day 3 with natamycin, relative to the control, there was an 84 and 77% reduction in the total microbial and fungal CFUs, respectively. In comparison, iodine (50 ppm) resulted in a more drastic reduction (79 and 93%, respectively) in the microbial and fungal counts immediately following treatment, but this effect was diminished on day 3. These results suggest that iodine more rapidly kills microbes (*cf.* natamycin) but is either consumed or there is a subpopulation of microbes that are tolerant and grow out with time. Despite the broad-spectrum antimicrobial activity of iodine, there are a number of microorganisms that display relatively high tolerances, including certain Penicillium and Fusarium species [34]. Therefore, the concentration of iodine was increased to 500 ppm to assess if it could prevent microbial growth, even though these concentrations would not be permissible in food products or animal feed. At this concentration, the microbial and fungal counts were immediately reduced by 99%, but despite a three-log reduction on day 3 (*cf.* control), there were still significant numbers of CFUs.

### 2.4. Treatment of Apple Pomace with Polysaccharides

**Figure 2:**
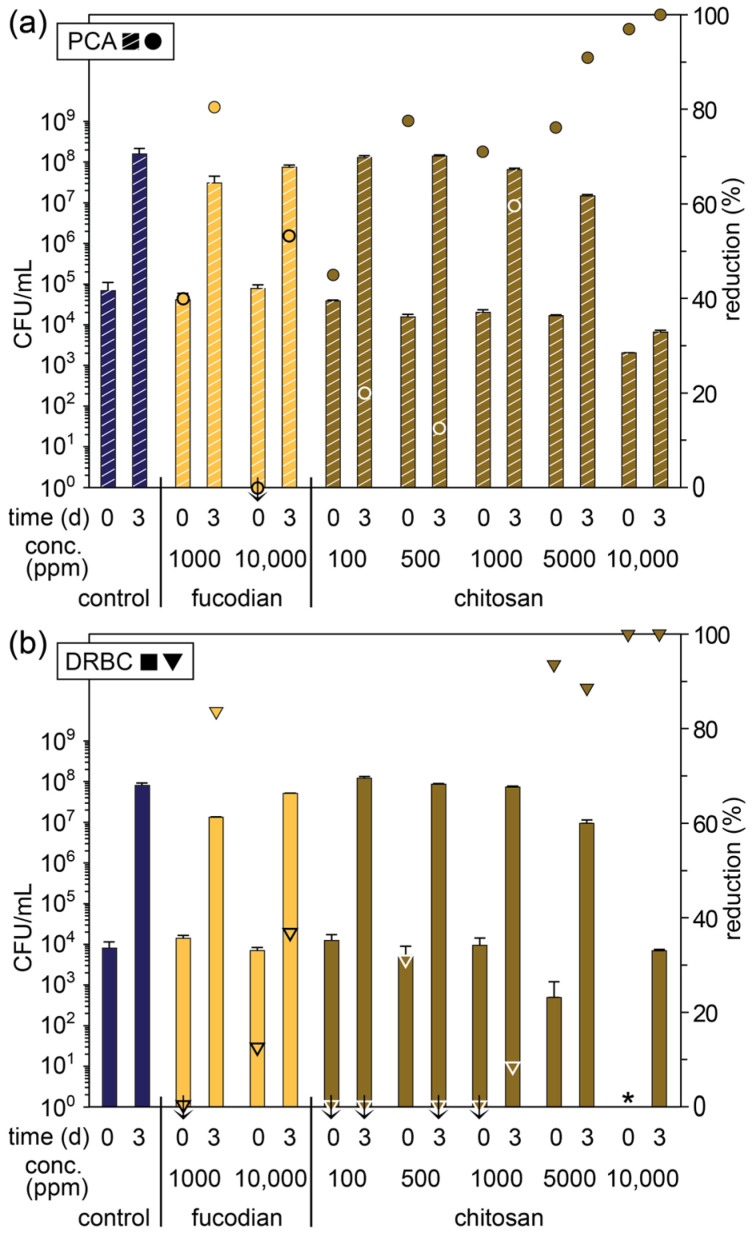
Microbial count (primary axis; bars) of fresh apple pomace (control) and pomace treated with different concentrations of fucoidan and chitosan plated on (**a**) PCA and (**b**) DRBC plates on day 0 (day of pressing) and day 3 (stored at ambient temperature) and percentage reduction in microbial count relative to the control on that day (secondary axis; symbols). * Below detectable limits. Symbols marked with a ↓ indicate an increase in the microbial count relative to the control.

Fucoidan and chitosan were selected as representative polysaccharides with reported bacteriostatic or bactericidal activity in the range of 1000 to 10,000 ppm [18,35,36,37]. In addition, chitosan has been widely reported to reduce post-harvest diseases on fruit and vegetables caused by pathogenic fungi, although typically when applied as a coating [38]. While fucoidan and chitosan are both polyelectrolytes, they would be oppositely charged in apple pomace (pH~3.4), providing an interesting comparison. Fucoidan is readily soluble in aqueous solutions across a wide pH range as the sulfonate groups remain negatively charged even at low pH values. In contrast, chitosan is only soluble under acidic conditions following protonation and is typically dissolved in dilute hydrochloric or acetic acid. In this study, a 10,000 ppm chitosan stock solution was prepared in 0.1 M acetic acid, and a series of dilutions were tested with the apple pomace (Figure 2 and SI, Tables S3 and S4).

At lower concentrations (100, 500, and 1000 ppm), chitosan was ineffective at preventing fungal growth on day 0 and 3 relative to the control, and in most cases promoted growth, presumably as a nitrogen source. Nevertheless, there was a decrease in the overall microbial counts, leading to modest reductions (45–78%) immediately on day 0, which further diminished on day 3. These results suggest that at these lower concentrations, chitosan has more of an impact on the bacterial species. At higher concentrations (5000 and 10,000 ppm), chitosan had a significant effect on the total microbial and fungal counts at both day 0 and 3. While reductions of > 88% were observed at a chitosan concentration of 5000 ppm, no fungal species were detected on day 0 with a concentration of 10,000 ppm, and there was a 97% reduction in microbial CFUs. By day 3, there was some outgrowth, but relative to the control, there was a > four-log reduction in both the total microbial and fungal CFUs. These results are consistent with previous studies indicating that chitosan has broad-spectrum antimicrobial activity due to its ability to electrostatically interact with negatively charged bacteria and fungi cell membranes, leading to permeabilization and disruption [38,39]. Nevertheless, it is possible that acetic acid also plays a role in inhibiting microbial growth (vide infra), although at the highest chitosan concentration, it only caused a slight decrease in the apple pomace pH from 3.4 to 3.3.

Compared to chitosan, fucoidan was significantly less effective at preventing microbial growth at both the concentrations tested. At the lower concentration of 1000 ppm, fucoidan was found to promote fungal growth while leading to a small reduction (40%) in the total microbial count on day 0. However, on day 3, the total microbial and fungal CFUs were both reduced by ~80% (Figure 2 and SI, Tables S3 and S4). In comparison, a higher concentration of fucoidan (10,000 ppm) promoted an increase in the total microbial count while having a negligible effect on the fungal count on day 0 and caused a moderate reduction in both (53 and 37%, respectively) on day 3. It is not immediately obvious why a lower concentration of fucoidan would lead to a greater reduction on day 3, although interactions between the fucoidan and the pomace matrix may play a role, which would require further investigation.

### 2.5. Treatment of Apple Pomace with Chitosan/Antimicrobial Combinations

**Figure 3:**
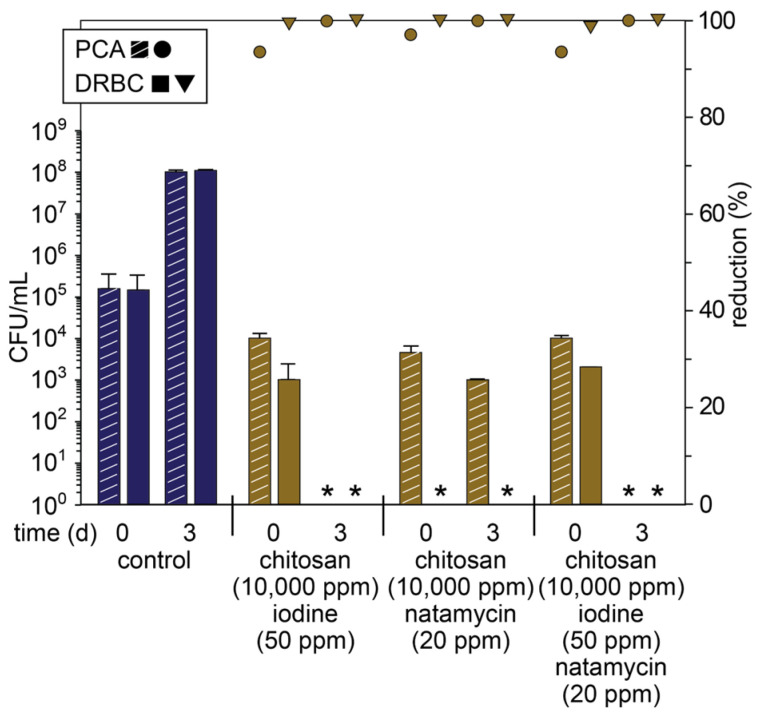
Microbial count (primary axis; bars) of fresh apple pomace (control) and pomace treated with different combinations of antimicrobials and chitosan plated on PCA and DRBC plates on day 0 (day of pressing) and day 3 (stored at ambient temperature) and percentage reduction in microbial count relative to the control on that day (secondary axis; symbols). * Below detectable limits.

With high concentrations of chitosan showing good antimicrobial activity, we proceeded to investigate synergistic effects between chitosan and iodine or natamycin. The addition of chitosan (10,000 ppm) and iodine (50 ppm) to the blended apple pomace resulted in a large reduction in the total microbial and fungal counts on day 0 (94 and 99%, respectively), and on day 3, no microorganisms were detected (Figure 3 and SI, Tables S5 and S6). Interestingly, this sustained activity is different from when chitosan or iodine were used separately (Figure 1 and Figure 2, respectively), implying that the interaction between chitosan and iodine modifies their antimicrobial activity. Indeed, iodophors prepared through the electrostatic interactions between protonated chitosan and iodine as the triiodide anion have been extensively characterized [40] and reported for applications in wound healing [41,42], providing a gradual release of iodine. Thus, the chitosan–triiodide complex is believed to provide a more persistent antimicrobial effect in the apple pomace, leading to a greater reduction in CFUs on day 3.

The addition of chitosan (10,000 ppm) and natamycin (20 ppm) to the blended apple pomace had a very similar effect to chitosan alone on day 0, including a large reduction (97%) in the total microbial count and the absence of detectable fungal species. However, on day 3, fungal species remained below detectable limits, and there was a five-log reduction in the total microbial CFUs. These results are consistent with the individual activities of chitosan and natamycin, although there is an obvious synergy that leads to enhanced activity over longer periods. Combining iodine (50 ppm) and natamycin (20 ppm) with chitosan (10,000 ppm) had an almost identical effect to the chitosan–iodine combination at both day 0 and 3 (Figure 3). Interestingly, unlike the chitosan–natamycin combination, the addition of iodine and natamycin prevented the immediate elimination of fungal species on day 0, which was attributed to the formation of the chitosan–iodine complex. While this complex evidently provides a longer-lasting antimicrobial effect, in the presence of natamycin, it inhibits the synergistic effects of chitosan and natamycin, possibly through modification of chitosan’s mode of action.

Despite the promising antimicrobial activity of chitosan and the chitosan combinations with iodine and natamycin, it is worth noting that high doses of chitosan are required. Not only is this likely cost-prohibitive, but the prolonged ingestion of large doses of chitosan may have adverse metabolic consequences. Although studies have shown that chitosan can lower plasma cholesterol and improve high-density lipoprotein cholesterol balance, it can also entrap nutrients in the intestinal tract and may alter calcium metabolism and the normal intestinal flora [43].

### 2.6. Influence of pH on Microbial Growth in Apple Pomace

**Figure 4:**
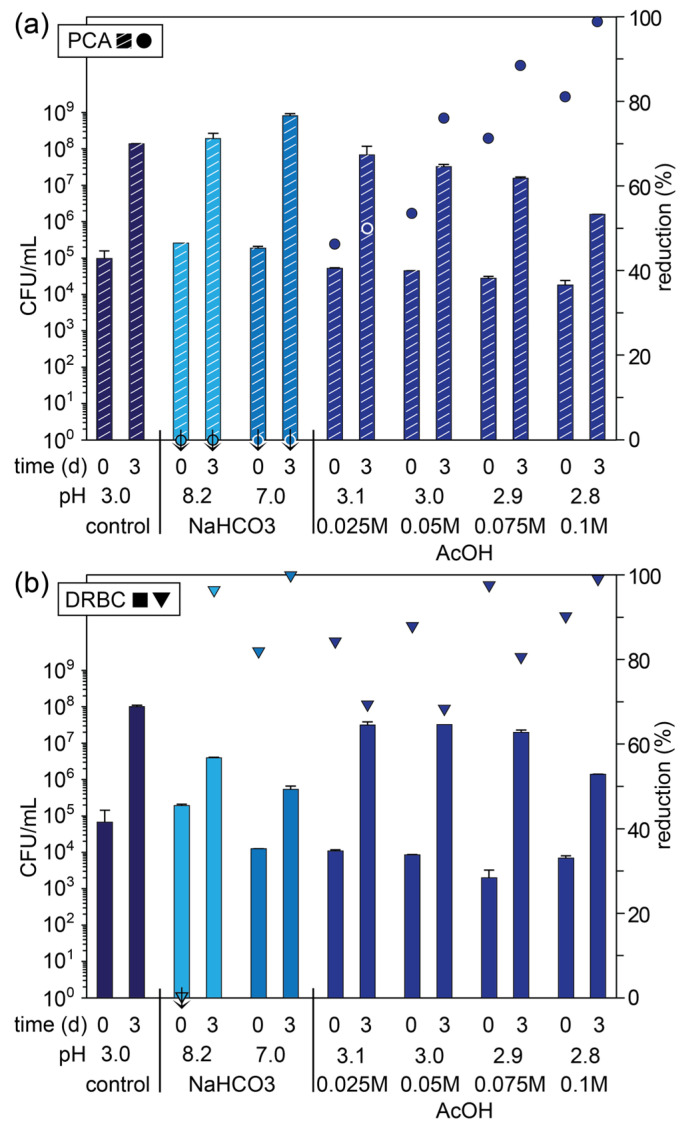
Microbial count (primary axis; bars) of fresh apple pomace (control) and pomace treated at different pH values plated on (**a**) PCA and (**b**) DRBC plates on day 0 (day of pressing) and day 3 (stored at ambient temperature) and percentage reduction in microbial count relative to the control on that day (secondary axis; symbols). Symbols marked with a ↓ indicate an increase in the microbial count relative to the control.

As previously mentioned, the apple pomace had a nominal pH of 3.4, and this was consistent across multiple batches. A comparison of the microbial and fungal CFUs for untreated apple pomace (control) on day 0 and 3 suggests that 70–75% of the CFUs are fungal species, ignoring competitive factors that may exist on the mixed culture PCA plates (Figure 4 and SI, Tables S7 and S8). This is consistent with the higher tolerance of many fungal species to wide pH ranges and extremes [44]. To assess the effect of elevated pH levels on microbial growth, blended apple pomace was pH-adjusted to 7 and 8.5. At pH 7, there was a large increase in microbial counts on day 0 and 3, accompanied by a reduction (>80%) in fungal counts, suggesting a more favorable environment for bacterial growth. Similar results were also observed at pH 8.5, particularly on day 3. Different microbes thrive under specific pH conditions, as has been demonstrated extensively with single and mixed cultures. For example, Rousk et al. have shown that bacterial and fungal growth were negatively correlated between pH 4.5 and 8.3, with lower pH values favoring fungal growth [45]. In apple pomace, which has a complex and diverse assortment of endogenous microorganisms, it is evident that different pH conditions favor the outgrowth of different genera and species [46]. In the present study, *Pseudomonas*, *Pantoea,* and *Rahnella* bacterial species would be expected to favor more neutral conditions; however, they have also been shown to be tolerant of lower pH conditions [47]. In comparison, *Hanseniaspora* and *Metschnikowia* yeasts and other fungal species (e.g., *Penicillium*) typically display optimal growth under mildly acidic pH conditions but are more tolerant of a wide range of pH conditions [48].

To assess the effect of more acidic conditions, and specifically, the influence of the acetic acid as in the chitosan solutions, blended apple pomace was treated with a series of acetic acid solutions ranging in concentration from 0.025 to 0.1 M (Figure 4 and SI, Tables S7 and S8). While all concentrations of acetic acid resulted in moderate to high reductions (46–99%) in both the total microbial and fungal CFUs, several trends emerged. An increase in the concentration resulted in a decrease in microbial and fungal counts on day 0. Interestingly, on day 3, there was a further reduction in the total microbial CFUs, but there was a diminished effect on the fungal counts as compared to day 0, with the exception of the highest concentration (0.1 M). While these results are consistent with the higher tolerance of fungal species to lower and sustained pH extremes [44], they also demonstrate the potential effectiveness of acetic acid at reducing the microbial load and spoilage. From comparison of the 10,000 ppm chitosan solution in 0.1 M acetic acid and the 0.1 M acetic acid solution (Figure 2 and Figure 4, respectively), it is evident that the significant antimicrobial activity of the former, particularly with respect to fungal species, results predominantly from chitosan. However, from a cost perspective, the preservation of apple pomace with relatively high concentrations of chitosan would likely be prohibitive.

### 2.7. Influence of Acetic Acid on Microbial Growth in Apple Pomace

**Figure 5:**
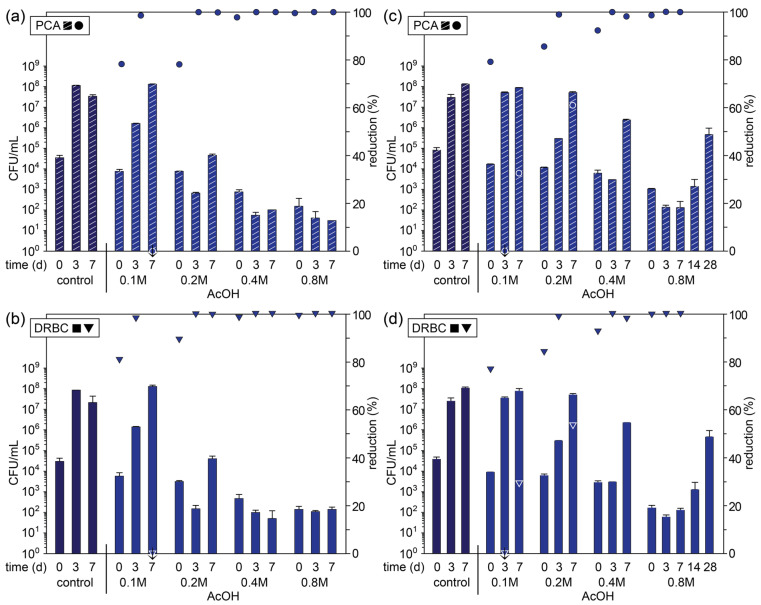
Microbial count (primary axis; bars) of (**a**,**b**) blended and (**c**,**d**) whole fresh apple pomace (control) and pomace treated with acetic acid solutions plated on (**a**,**c**) PCA and (**b**,**d**) DRBC plates on day 0 (day of pressing) and days following storage at ambient temperature and percentage reduction in microbial count relative to the control on that day (secondary axis; symbols). Symbols marked with a ↓ indicate an increase in the microbial count relative to the control.

Given that acetic acid alone showed favorable results, we considered the prospect of using more concentrated solutions and extending our studies over a longer period. Therefore, the blended apple pomace was initially treated with acetic acid at concentrations ranging from 0.1 to 0.8 M over 7 days (Figure 5a,b, and SI, Tables S9 and S10), with the highest concentration being approximately equivalent to commercially available vinegar solutions (4–5%). With increasing concentration, there was a significant reduction in the total microbial and fungal CFUs on days 0, 3, and 7 relative to the control. For 0.1 and 0.2 M acetic acid (pH 3.2 and 3.1, respectively), there was evidence for recovery and outgrowth on day 7, whereas the microbial and fungal counts remained consistently low at concentrations of 0.4 and 0.8 M (pH 3.0 and 2.8, respectively), with > five-log reductions. While it is likely that the pH of the solutions is predominantly responsible for the inhibition of the microbes and reduced microbial load, acetic acid may also display some inherent toxicity to specific species that is accentuated with increasing concentration. For example, Stratford et al. have shown that different weak acids with the same *pk_a_* display very different toxicities toward fungal species, although acetic acid inhibition was predominantly found to result from cytoplasmic acidification [49]. This may suggest that other weak acids are also suitable for the preservation of apple pomace, although further tests would be required to assess their potential effectiveness against the varied fungal species.

The homogenization and dilution of the apple pomace with the antimicrobial agent or preservative via blending allowed experiments to be conducted consistently and reproducibly. However, at scale, this approach is unlikely to be feasible for the storage and/or transportation of apple pomace prior to processing and would introduce a later filtration step to concentrate the pomace. A more feasible approach involves simply submerging the whole apple pomace in a container with the minimum amount of preservative, which can be syphoned off later as required. Therefore, we tested the effectiveness of acetic solutions (0.1 to 0.8 M) in preventing microbial growth in raw, unprocessed apple pomace through submersion (~3 kg/L). In general, a similar trend was observed for the submerged pomace as compared to the blended pomace, with an increase in concentration leading to an overall reduction in microbial and fungal growth, although to a lesser extent, particularly at acetic acid concentrations below 0.8 M, where there was relatively significant microbial and fungal counts by day 7 (Figure 5c,d, and SI, Tables S11 and S12).

For the 0.8 M acetic acid solution, the microbial and fungal counts remained very low out to day 7 (>five-log reduction relative to the control), and therefore, the experiment was extended out to 28 days. On day 14, there was a small increase in the CFUs compared to day 7, but by day 28, there was significant outgrowth of the surviving microorganisms, which at this point appeared to be predominantly fungal species, with the microbial and fungal counts being equivalent. These results suggest that 0.8 M acetic acid, and by extension, commercial vinegar, is effective at reducing the microbial load and significantly inhibiting microbial growth in apple pomace over a period of at least 14 d, and at low doses on a weight-to-volume basis. It is this low dosing that is likely responsible for the lower concentrations being less effective with the submerged pomace (*cf.* blended pomace), as well as the restricted permeation of acetic acid into the pomace cake harboring microbes.

Acetic acid has been used extensively throughout history as a preservative, beneficial food additive, and condiment [50]. As demonstrated in this study, acetic acid is highly effective at inhibiting microbial growth in apple pomace, and compared to other additives or combinations, is a relatively cost-effective alternative. Furthermore, following storage and/or transportation, the acetic acid solution could potentially be separated and upcycled (e.g., apple cider vinegar). The use of commercial-strength acetic acid (i.e., vinegar) for preservation would minimize the need for specialized handling or processing equipment, making processes for managing large volumes safe and practical. While the apple pomace is likely to retain some residual acetic acid following separation, this may actually be beneficial for particular applications, including animal feed for ruminants. Acetic acid is considered an essential nutrient for ruminants and is often supplemented in their feed, with studies showing that the production of milk and milk fat in dairy cows correlated linearly with acetic acid intake [51]. Furthermore, the European Food Safety Authority promotes the use of acetic acid as a preservative for feed and has set no limits for ruminants [52]. Nevertheless, the storage of apple pomace in acetic acid may result in the dissolution and leaching of soluble sugars and bioactives, resulting in a change in the nutritional profile that would need to be explored further.

### 2.8. Fermentation of Acetic Acid Preserved Apple Pomace

**Figure 6:**
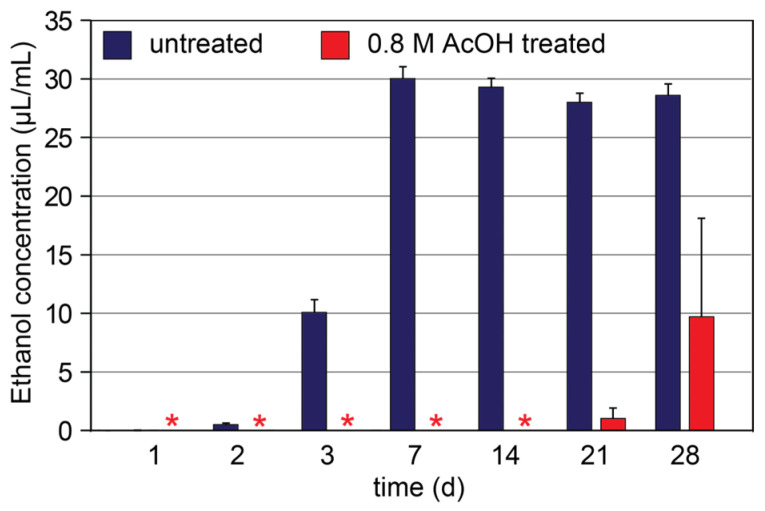
Ethanol concentration as a function of time for fresh apple pomace and pomace treated with 0.8 M acetic acid (*n* = 4). * Below detectable limits. Error bars are large for acetic-acid-treated samples as no ethanol was detected in 2 out of 4 replicates at any time point.

Untreated, apple pomace spoilage can occur rapidly over several days, resulting in mold growth and fermentation, and the generation of significant amounts of ethanol that can be undesirable in the upcycling of pomace, particularly as animal feed. Therefore, any intentional preservation approach should aim to reduce or eliminate microbial growth and ethanol production. To determine if acetic acid was sufficient in preventing fermentation, the ethanol concentration was measured via gas chromatography for pomace submerged in acetic acid (0.8 M) versus water (Figure 6). In water, significant amounts of ethanol were noted on day 3 and reached an equilibrium on day 7, presumably due to the complete consumption of digestible free sugars. In comparison, no ethanol was detected in the acetic-acid-treated pomace until day 21, further supporting the effectiveness of acetic acid as a preservative.

## 3. Conclusions

Of the preservatives tested, chitosan and combinations of chitosan with iodine and natamycin were found to be highly effective at inhibiting the growth of microorganisms in apple pomace. However, high concentrations of chitosan were required, which would likely be cost-prohibitive and may have adverse metabolic consequences if consumed regularly. From a cost and safety perspective, acetic acid was by far the most efficacious preservative and could also be easily deployed at scale with minimal processing requirements. Overall, 0.8 M acetic acid, approximately equivalent to commercial vinegar, was sufficient to reduce the microbial load and significantly retard microbial growth over 14 days at relatively low doses and prevent fermentation and the formation of ethanol. Although more studies are required to determine the optimal dose of acetic acid, and further reductions may be possible, the amount used in this study for the preservation of whole pomace would not contribute significantly to its mass, and therefore, transportation costs. Further studies are required to assess the feasibility of draining or decanting the acetic acid solution from the pomace at scale as required and determine the level of residual acetic acid that remains in the pomace. In some products or applications, residual acetic acid may be desirable, particularly in feed for ruminants, which can increase milk production yields. In addition, separation of the acetic acid may also provide opportunities to isolate micronutrients (e.g., polyphenolics), upcycle the extract as apple cider vinegar, or simply reuse the acetic acid after sterilization. Future studies are also required to study the impact of acetic acid preservation on the nutritional profile of apple pomace.

## 4. Experimental

### 4.1. Materials

Chitosan (medium molecular weight, 75–85% deacylated, 200–800 cP, 1% *w*/*v* in 1% *v*/*v* acetic acid), natamycin (pimaricin, 95%), and peptone were purchased from Merck (Sigma-Aldrich, Sydney, Australia). Glacial acetic acid (Analytical Reagent (AR) grade), iodine (AR grade), potassium iodide (AR grade), and sodium hydrogen carbonate (AR grade) were purchased from ChemSupply (Adelaide, Australia). *Fucus vesiculosus* (fucoidan) extract was supplied by Marinova, Hobart, Australia.

Fresh apple pomace consisting of a mixture of Pink Lady, Granny Smith, Royal Gala, and Kanzi varieties was provided by Ashton Valley Fresh (Ashton, SA, Australia), stored in a sealed plastic bag, and used within 6 h of pressing. Oven drying (70 °C, 24 h) of the pomace from multiple batches provided a solids content of 25.9 ± 0.5% w/w.

Ultrapure water (resistivity > 18 MΩ.cm) was obtained from a Sartorius Arium Pro Ultrapure Water System. pH calibration standards (1.68, 4.01, 7.01, and 10.01) were purchased from Hanna Instruments (Smithfield, VA, USA). All chemicals were used as received unless otherwise specified.

### 4.2. Methods

Preparation of the blended apple pomace samples: Fresh apple pomace (100 g) and water (300 g) were combined in a blender (MultiBlender Platinum, Sunbeam, Boca Raton, FL, USA) and blended at ~20 krpm (the highest setting) for 3 min (30 s on, 10 s off). The resulting mixture was transferred into a sealed plastic container and stored at ambient temperature (~22 °C) prior to microbiological testing. Aliquots were removed immediately for day 0 microbiological testing. For treated pomace samples, the selected preservatives were initially prepared in water prior to blending with the apple pomace. The concentration of the preservatives was calculated with respect to just the wet pomace and not the additional water.

Preparation of the whole apple pomace samples: Fresh apple pomace (300 g) and water or acetic acid solutions (100 mL) were combined in a plastic container and stored at ambient temperature (~22 °C) prior to microbiological testing. Aliquots were removed immediately for day 0 microbiological testing.

Microbiological testing: Aliquots (10 g) of blended pomace samples or whole pomace were weighed in a stomacher bag, and 0.1% *w*/*v* peptone (90 mL) was added. The bag was placed in a stomacher (Stomacher 400 Circulator, Seward, Worthing, UK) and homogenized for 2 min. Serial dilutions were then prepared with water covering four or five orders of magnitude. Aliquots (100 µL) from each dilution were then spread on either PCA or DRBC plates (Thermo Fisher, Adelaide, Australia) in triplicate. The plates were stored at 25 °C, and the number of CFUs were manually counted periodically.

Identification of microorganisms: Individual species of microorganisms were identified using a Bruker MALDI Biotyper Sirius system (Billerica, MA, USA). In addition, a microscope (CX33 Biological Microscope, Olympus, Melbourne, Australia) was used to identify the genus of microorganisms.

## Data Availability

All plates counts are provided in the Supplementary Materials.

## Supplementary Materials

The following supporting information can be downloaded at https://www.mdpi.com/article/10.3390/foods14142438/s1. Figure S1: Change in pH as a function of time for treated apple pomace; Table S1: Average microbial counts on PCA plates following treatment of blended apple pomace with natamycin and iodine; Table S2: Average microbial counts on DRBC plates following treatment of blended apple pomace with natamycin and iodine; Table S3: Average microbial counts on PCA plates following treatment of blended apple pomace with fucoidan and chitosan; Table S4: Average microbial counts on DRBC plates following treatment of blended apple pomace with fucoidan and chitosan; Table S5: Average microbial counts on PCA plates following treatment of blended apple pomace with chitosan combinations; Table S6: Average microbial counts on DRBC plates following treatment of blended apple pomace with chitosan combinations; Table S7: Average microbial counts on PCA plates following treatment of blended apple pomace at different pH values; Table S8: Average microbial counts on DRBC plates following treatment of blended apple pomace at different pH values; Table S9: Average microbial counts on PCA plates following treatment of blended apple pomace with 0.1–0.8 M acetic acid; Table S10: Average microbial counts on DRBC plates following treatment of blended apple pomace with 0.1–0.8 M acetic acid; Table S11: Average microbial counts on PCA plates following treatment of whole apple pomace with 0.1–0.8 M acetic acid; Table S12: Average microbial counts on DRBC plates following treatment of whole apple pomace with 0.1–0.8 M acetic acid.
