# Supplementary material for: Comparison of Preservatives for the Prevention of Microbial Spoilage of Apple Pomace During Storage
Source: Foods. 2025 Jul 10;14(14):2438. doi: 10.3390/foods14142438 (PMC12294221; doi:10.3390/foods14142438)
Supplement: Supplementary file 1 [file foods-14-02438-s001.zip › foods-3706900-supplementary.pdf]

SUPPLEMENTARY INFORMATION

Comparison of preservatives for the prevention of microbial spoilage of apple pomace during storage

Ashley Harratt,[1,2] Wenyuan Wu,[1,2] Peyton Strube,[1] Joseph Ceravolo Jr.,[3] David Beattie,[2] Tara Pukala,[4] Marta Krasowska,[2,*] Anton Blencowe[1,*]

[1] Applied Chemistry and Translational Biomaterials (ACTB) Group, Centre for Pharmaceutical Innovation (CPI), UniSA Clinical and Health Sciences, University of South Australia, Adelaide, SA 5000, Australia.

[2] Future Industries Institute (FII), UniSA Science, Technology, Engineering and Mathematics (STEM), University of South Australia, Mawson Lakes, SA 5098, Australia.

[3] Ashton Valley Fresh, Lobethal Rd, Ashton, SA 5137, Australia.

[4] School of Physics, Chemistry and Earth Sciences, University of Adelaide, Adelaide, SA 5005, Australia.

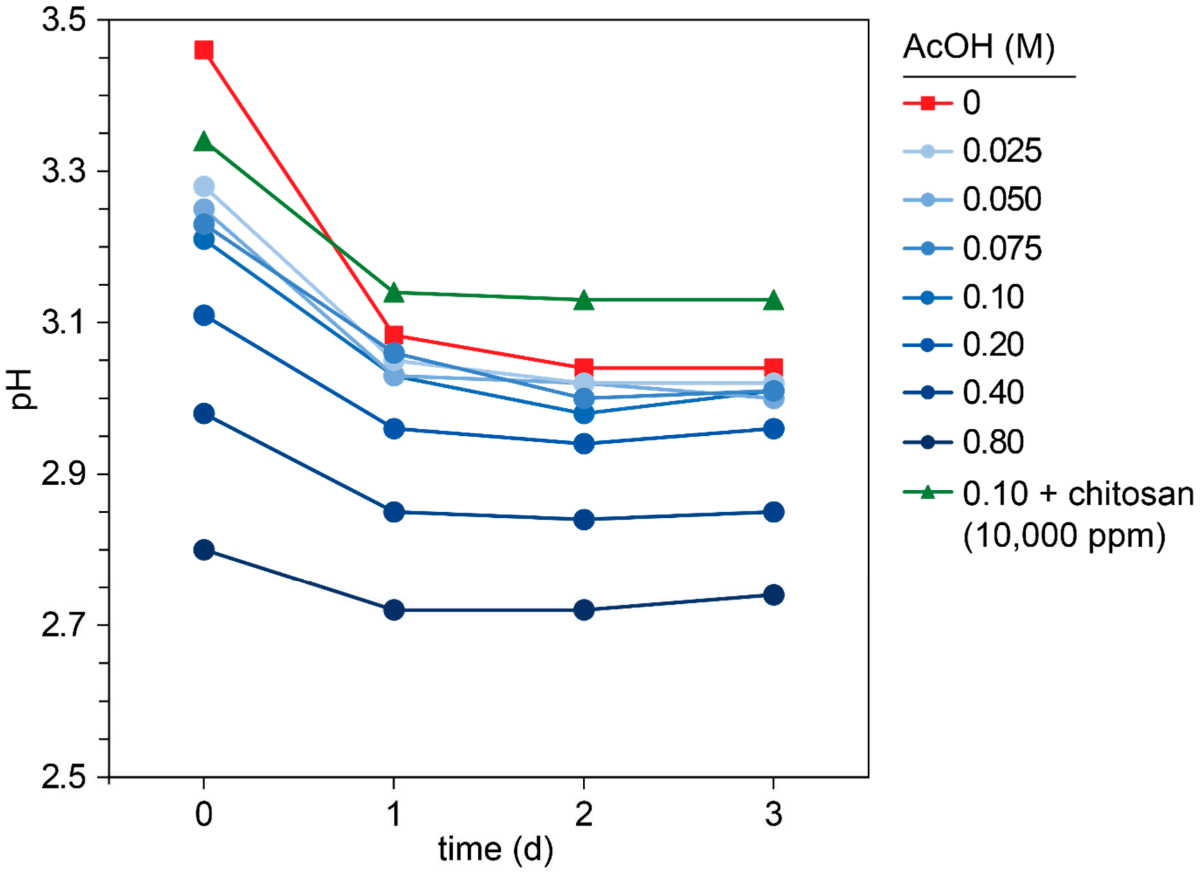

Figure S1: Change in pH as a function of time for blended apple pomace and apple pomace treated with acetic acid solutions (0.025-0.8 M) or chitosan in acetic acid (0.1 M). Symbols represent data points and lines are to guide the eye.

Table S1: Average microbial counts on PCA plates following treatment of blended apple pomace with natamycin and iodine, and change in microbial counts relative to the untreated control.

Treatment[a]	Day	Average microbial count ± std.dev (CFU/mL)	Log reduction[b]	% reduction[b]
Control	0	$2.2 \times 10^5 \pm 8.7 \times 10^4$		
	3	$1.4 \times 10^8 \pm 5.8 \times 10^7$		
Natamycin, 50 ppm	0	$2.4 \times 10^5 \pm 1.1 \times 10^5$	-0.04	-8.8
	3	$2.2 \times 10^7 \pm 6.0 \times 10^6$	0.80	84.3
Iodine, 50 ppm	0	$4.7 \times 10^4 \pm 3.5 \times 10^3$	0.68	78.9
	3	$4.7 \times 10^7 \pm 7.8 \times 10^6$	0.49	67.3
Iodine, 500 ppm	0	$2.0 \times 10^3 \pm 1.4 \times 10^3$	2.04	99.1
	3	$5.8 \times 10^4 \pm 4.9 \times 10^3$	3.39	>99.9

[a] apple pomace treatment, where control is no treatment. [b] Log reduction and % reduction of CFUs resulting from treatments relative to the control on that specific day.

Table S2: Average microbial counts on DRBC plates following treatment of blended apple pomace with natamycin and iodine, and change in microbial counts relative to the untreated control.

Treatment[a]	Day	Average microbial count ± std.dev (CFU/mL)	Log reduction[b]	% reduction[b]
Control	0	$1.8 \times 10^5 \pm 4.6 \times 10^4$		
	3	$7.0 \times 10^7 \pm 2.2 \times 10^7$		
Natamycin, 50 ppm	0	$9.8 \times 10^4 \pm 6.0 \times 10^4$	0.26	45.2
	3	$1.6 \times 10^7 \pm 5.9 \times 10^6$	0.63	76.7
Iodine, 50 ppm	0	$1.3 \times 10^4 \pm 1.4 \times 10^3$	1.14	92.7
	3	$3.8 \times 10^7 \pm 2.2 \times 10^6$	0.27	46.7
Iodine, 500 ppm	0	$1.5 \times 10^3 \pm 2.1 \times 10^2$	2.08	99.2
	3	$3.8 \times 10^4 \pm 2.8 \times 10^3$	3.27	>99.9

[a] apple pomace treatment, where control is no treatment. [b] Log reduction and % reduction of CFUs resulting from treatments relative to the control on that specific day.

Table S3: Average microbial counts on PCA plates following treatment of blended apple pomace with fucoidan and chitosan, and change in microbial counts relative to the untreated control.

Treatment[a]	Day	Average microbial count ± std.dev (CFU/mL)	Log reduction[b]	% reduction[b]
Control	0	$6.9 \times 10^4 \pm 3.8 \times 10^4$		
	3	$1.6 \times 10^8 \pm 5.3 \times 10^7$		
Fucoidan, 1,000 ppm	0	$4.2 \times 10^4 \pm 1.6 \times 10^4$	0.22	40.0
	3	$3.1 \times 10^7 \pm 1.3 \times 10^7$	0.71	80.5
Fucoidan, 10,000 ppm	0	$7.9 \times 10^4 \pm 1.5 \times 10^4$	-0.06	-13.6
	3	$7.5 \times 10^7 \pm 7.8 \times 10^6$	0.33	53.1
Chitosan, 100 ppm	0	$3.8 \times 10^4 \pm 1.4 \times 10^3$	0.26	45.0
	3	$1.3 \times 10^8 \pm 1.3 \times 10^7$	0.10	20.1
Chitosan, 500 ppm	0	$1.6 \times 10^4 \pm 2.1 \times 10^3$	0.65	77.6
	3	$1.4 \times 10^8 \pm 8.5 \times 10^6$	0.06	12.6
Chitosan, 1,000 ppm	0	$2.0 \times 10^4 \pm 2.8 \times 10^3$	0.54	71.1
	3	$6.5 \times 10^7 \pm 4.9 \times 10^6$	0.39	59.4
Chitosan, 5,000 ppm	0	$1.7 \times 10^4 \pm 7.1 \times 10^2$	0.62	76.1
	3	$1.4 \times 10^7 \pm 1.1 \times 10^6$	1.04	90.9
Chitosan, 10,000 ppm	0	$2.0 \times 10^3 \pm 1.0 \times 10^2$	1.54	97.1
	3	$6.5 \times 10^3 \pm 7.1 \times 10^2$	4.39	>99.9

[a] apple pomace treatment, where control is no treatment. [b] Log reduction and % reduction of CFUs resulting from treatments relative to the control on that specific day.

Table S4: Average microbial counts on DRBC plates following treatment of blended apple pomace with fucoidan and chitosan, and change in microbial counts relative to the untreated control.

Treatment[a]	Day	Average microbial count ± std.dev (CFU/mL)	Log reduction[b]	% reduction[b]
Control	0	$8.0 \times 10^3 \pm 3.6 \times 10^3$		
	3	$8.1 \times 10^7 \pm 1.2 \times 10^7$		
Fucoidan, 1,000 ppm	0	$1.4 \times 10^4 \pm 2.8 \times 10^3$	-0.24	-75.0
	3	$1.3 \times 10^7 \pm 5.7 \times 10^5$	0.79	83.6
Fucoidan, 10,000 ppm	0	$7.0 \times 10^3 \pm 1.4 \times 10^3$	0.06	12.5
	3	$5.1 \times 10^7 \pm 1.4 \times 10^6$	0.20	36.8
Chitosan, 100 ppm	0	$1.3 \times 10^4 \pm 4.9 \times 10^3$	-0.19	-56.3
	3	$1.2 \times 10^8 \pm 1.3 \times 10^7$	-0.18	-51.2
Chitosan, 500 ppm	0	$5.5 \times 10^3 \pm 3.5 \times 10^3$	0.16	31.3
	3	$8.7 \times 10^7 \pm 4.2 \times 10^6$	-0.03	-7.8
Chitosan, 1,000 ppm	0	$9.5 \times 10^3 \pm 4.9 \times 10^3$	-0.07	-18.8
	3	$7.4 \times 10^7 \pm 4.2 \times 10^6$	0.04	8.3
Chitosan, 5,000 ppm	0	$5.0 \times 10^2 \pm 7.1 \times 10^2$	1.20	93.8
	3	$9.5 \times 10^6 \pm 2.1 \times 10^6$	0.93	88.3
Chitosan, 10,000 ppm	0	---[c]	--- [c]	---[c]
	3	$7.0 \times 10^3 \pm 1.0 \times 10^2$	4.06	>99.9

[a] apple pomace treatment, where control is no treatment. [b] Log reduction and % reduction of CFUs resulting from treatments relative to the control on that specific day. [c] No microbes observed; below detection limits.

Table S5: Average microbial counts on PCA plates following treatment of blended apple pomace with chitosan combinations with iodine and natamycin, and change in microbial counts relative to the untreated control.

Treatment[a]	Day	Average microbial count ± std.dev (CFU/mL)	Log reduction[b]	% reduction[b]
Control	0	$1.5 \times 10^5 \pm 1.9 \times 10^5$		
	3	$1.0 \times 10^8 \pm 1.1 \times 10^7$		
Chitosan, 10,000 ppm; Iodine, 50 ppm	0	$1.0 \times 10^4 \pm 2.8 \times 10^3$	1.19	93.5
	3	---[c]	---[c]	---[c]
Chitosan, 10,000 ppm; Natamycin, 20 ppm	0	$4.5 \times 10^3 \pm 2.1 \times 10^3$	1.54	97.1
	3	$1.0 \times 10^3 \pm 1.0 \times 10^2$	5.00	>99.9
Chitosan, 10,000 ppm; Iodine, 50 ppm; Natamycin 20 ppm	0	$1.0 \times 10^4 \pm 1.4 \times 10^3$	1.19	93.5
	3	---[c]	---[c]	---[c]

[a] apple pomace treatment, where control is no treatment. [b] Log reduction and % reduction of CFUs resulting from treatments relative to the control on that specific day. [c] No microbes observed; below detection limits.

Table S6: Average microbial counts on DRBC plates following treatment of blended apple pomace with chitosan combinations with iodine and natamycin, and change in microbial counts relative to the untreated control.

Treatment[a]	Day	Average microbial count ± std.dev (CFU/mL)	Log reduction[b]	% reduction[b]
Control	0	$1.4 \times 10^5 \pm 1.9 \times 10^5$		
	3	$1.1 \times 10^8 \pm 7.5 \times 10^6$		
Chitosan, 10,000 ppm; Iodine, 50 ppm	0	$1.0 \times 10^3 \pm 1.4 \times 10^3$	2.15	99.3
	3	---[c]	---[c]	---[c]
Chitosan, 10,000 ppm; Natamycin, 20 ppm	0	---[c]	---[c]	---[c]
	3	---[c]	---[c]	---[c]
Chitosan, 10,000 ppm; Iodine, 50 ppm; Natamycin 20 ppm	0	$2.0 \times 10^3 \pm 1.0 \times 10^2$	1.85	98.6
	3	---[c]	---[c]	---[c]

[a] apple pomace treatment, where control is no treatment. [b] Log reduction and % reduction of CFUs resulting from treatments relative to the control on that specific day. [c] No microbes observed; below detection limits.

Table S7: Average microbial counts on PCA plates following treatment of blended apple pomace at different pH values, and change in microbial counts relative to the untreated control.

Treatment[a]	Day	Average microbial count ± std.dev (CFU/mL)	Log reduction[b]	% reduction[b]
Control	0	$9.6 \times 10^4 \pm 6.8 \times 10^4$		
	3	$1.4 \times 10^8 \pm 8.3 \times 10^6$		
pH 8.5	0	$2.5 \times 10^5 \pm 1.3 \times 10^4$	-0.42	-163.2
	3	$1.9 \times 10^8 \pm 8.7 \times 10^7$	-0.14	-39.3
pH 7.0	0	$1.8 \times 10^5 \pm 3.5 \times 10^4$	-0.28	-92.7
	3	$8.1 \times 10^8 \pm 1.5 \times 10^8$	-0.78	-497.1
pH 3.3, 0.025 M AcOH	0	$5.2 \times 10^4 \pm 4.9 \times 10^3$	0.27	46.2
	3	$6.8 \times 10^7 \pm 5.4 \times 10^7$	0.30	50.0
pH 3.3, 0.050 M AcOH	0	$4.5 \times 10^4 \pm 2.1 \times 10^3$	0.33	53.5
	3	$3.3 \times 10^7 \pm 6.4 \times 10^6$	0.62	76.1
pH 3.2, 0.075 M AcOH	0	$2.8 \times 10^4 \pm 4.9 \times 10^3$	0.54	71.3
	3	$1.6 \times 10^7 \pm 1.8 \times 10^6$	0.94	88.5
pH 3.2, 0.10 M AcOH	0	$1.8 \times 10^4 \pm 7.1 \times 10^3$	0.73	81.2
	3	$1.6 \times 10^6 \pm 7.8 \times 10^4$	1.94	98.8

[a] apple pomace treatment, where control is no treatment. [b] Log reduction and % reduction of CFUs resulting from treatments relative to the control on that specific day.

Table S8: Average microbial counts on DRBC plates following treatment of blended apple pomace at different pH values, and change in microbial counts relative to the untreated control.

Treatment[a]	Day	Average microbial count ± std.dev (CFU/mL)	Log reduction[b]	% reduction[b]
Control	0	$6.8 \times 10^4 \pm 8.4 \times 10^4$		
	3	$1.0 \times 10^8 \pm 1.5 \times 10^7$		
pH 8.5	0	$1.9 \times 10^5 \pm 2.8 \times 10^4$	-0.46	-187.1
	3	$4.0 \times 10^6 \pm 3.5 \times 10^5$	1.41	96.1
pH 7.0	0	$1.3 \times 10^4 \pm 7.1 \times 10^2$	0.73	81.5
	3	$5.4 \times 10^5 \pm 1.6 \times 10^5$	2.27	99.5
pH 3.3, 0.025 M AcOH	0	$1.1 \times 10^4 \pm 1.4 \times 10^3$	0.79	83.8
	3	$3.2 \times 10^7 \pm 9.2 \times 10^6$	0.51	68.9
pH 3.3, 0.050 M AcOH	0	$8.5 \times 10^3 \pm 7.1 \times 10^2$	0.90	87.5
	3	$3.3 \times 10^7 \pm 7.1 \times 10^5$	0.49	68.0
pH 3.2, 0.075 M AcOH	0	$2.0 \times 10^3 \pm 1.4 \times 10^3$	1.53	97.0
	3	$2.0 \times 10^7 \pm 4.3 \times 10^6$	0.71	80.3
pH 3.2, 0.10 M AcOH	0	$7.0 \times 10^3 \pm 1.4 \times 10^3$	0.99	89.7
	3	$1.4 \times 10^6 \pm 7.8 \times 10^4$	1.86	98.6

[a] apple pomace treatment, where control is no treatment. [b] Log reduction and % reduction of CFUs resulting from treatments relative to the control on that specific day.

Table S9: Average microbial counts on PCA plates following treatment of blended apple pomace with 0.1–0.8 M acetic acid (AcOH), and change in microbial counts relative to the untreated control.

Treatment[a]	Day	Average microbial count ± std.dev (CFU/mL)	Log reduction[b]	% reduction[b]
Control	0	$3.5 \times 10^4 \pm 1.1 \times 10^4$		
	3	$1.1 \times 10^8 \pm 4.9 \times 10^6$		
	7	$3.4 \times 10^7 \pm 6.4 \times 10^6$		
pH 3.2, 0.10 M AcOH	0	$7.5 \times 10^3 \pm 1.8 \times 10^3$	0.66	78.3
	3	$1.6 \times 10^6 \pm 7.8 \times 10^4$	1.85	98.6
	7	$1.3 \times 10^8 \pm 5.7 \times 10^6$	-0.59	-285.1
pH 3.1, 0.20 M AcOH	0	$7.6 \times 10^3 \pm 2.1 \times 10^2$	0.66	78.1
	3	$6.5 \times 10^2 \pm 7.1 \times 10^1$	5.23	>99.9
	7	$4.5 \times 10^4 \pm 7.1 \times 10^3$	2.87	99.9
pH 3.0, 0.40 M AcOH	0	$7.5 \times 10^2 \pm 2.1 \times 10^2$	1.66	97.8
	3	$5.5 \times 10^1 \pm 2.1 \times 10^1$	6.31	>99.9
	7	$1.0 \times 10^2 \pm 1.0 \times 10^1$	5.53	>99.9
pH 2.8, 0.80 M AcOH	0	$1.5 \times 10^2 \pm 2.1 \times 10^2$	2.36	99.6
	3	$4.0 \times 10^1 \pm 4.2 \times 10^1$	6.45	>99.9
	7	$3.0 \times 10^1 \pm 0$	6.05	>99.9

[a] apple pomace treatment, where control is no treatment. [b] Log reduction and % reduction of CFUs resulting from treatments relative to the control on that specific day.

Table S10: Average microbial counts on DRBC plates following treatment of blended apple pomace with 0.1–0.8 M acetic acid (AcOH), and change in microbial counts relative to the untreated control.

Treatment[a]	Day	Average microbial count ± std.dev (CFU/mL)	Log reduction[b]	% reduction[b]
Control	0	$3.0 \times 10^4 \pm 1.3 \times 10^4$		
	3	$8.5 \times 10^7 \pm 7.1 \times 10^5$		
	7	$2.1 \times 10^7 \pm 2.3 \times 10^7$		
pH 3.2, 0.10 M AcOH	0	$5.8 \times 10^3 \pm 2.7 \times 10^3$	0.71	80.7
	3	$1.4 \times 10^6 \pm 7.8 \times 10^4$	1.78	98.3
	7	$1.3 \times 10^8 \pm 2.1 \times 10^7$	-0.78	-509.5
pH 3.1, 0.20 M AcOH	0	$3.2 \times 10^3 \pm 3.5 \times 10^2$	0.98	89.5
	3	$1.5 \times 10^2 \pm 7.1 \times 10^1$	5.75	>99.9
	7	$4.0 \times 10^4 \pm 1.4 \times 10^4$	2.72	99.8
pH 3.0, 0.40 M AcOH	0	$4.8 \times 10^2 \pm 2.6 \times 10^2$	1.80	98.4
	3	$1.0 \times 10^2 \pm 2.8 \times 10^1$	5.93	>99.9
	7	$5.0 \times 10^1 \pm 7.1 \times 10^1$	5.62	>99.9
pH 2.8, 0.80 M AcOH	0	$1.4 \times 10^2 \pm 5.7 \times 10^1$	2.33	99.5
	3	$1.1 \times 10^2 \pm 1.4 \times 10^1$	5.89	>99.9
	7	$1.4 \times 10^2 \pm 4.2 \times 10^1$	5.18	>99.9

[a] apple pomace treatment, where control is no treatment. [b] Log reduction and % reduction of CFUs resulting from treatments relative to the control on that specific day.

Table S11: Average microbial counts on PCA plates following treatment of whole apple pomace with 0.1–0.8 M acetic acid (AcOH), and change in microbial counts relative to the untreated control.

Treatment[a]	Day	Average microbial count ± std.dev (CFU/mL)	Log reduction[b]	% reduction[b]
Control	0	$8.0 \times 10^4 \pm 2.8 \times 10^4$		
	3	$3.0 \times 10^7 \pm 1.2 \times 10^7$		
	7	$1.3 \times 10^8 \pm 6.4 \times 10^6$		
pH 3.2, 0.10 M AcOH	0	$1.7 \times 10^4 \pm 6.4 \times 10^2$	0.68	79.2
	3	$5.1 \times 10^7 \pm 4.5 \times 10^6$	-0.24	-73.6
	7	$8.8 \times 10^7 \pm 2.8 \times 10^6$	0.17	32.6
pH 3.1, 0.20 M AcOH	0	$1.2 \times 10^4 \pm 6.4 \times 10^2$	0.84	85.6
	3	$3.0 \times 10^5 \pm 1.0 \times 10^4$	1.99	99.0
	7	$5.1 \times 10^7 \pm 5.7 \times 10^6$	0.41	60.9
pH 3.0, 0.40 M AcOH	0	$6.2 \times 10^3 \pm 2.5 \times 10^3$	1.11	92.3
	3	$3.0 \times 10^3 \pm 1.0 \times 10^2$	3.99	>99.9
	7	$2.4 \times 10^6 \pm 2.1 \times 10^5$	1.74	98.2
pH 2.8, 0.80 M AcOH	0	$1.1 \times 10^3 \pm 7.1 \times 10^1$	1.88	98.7
	3	$1.4 \times 10^2 \pm 3.5 \times 10^1$	5.34	>99.9
	7	$1.3 \times 10^2 \pm 1.3 \times 10^2$	6.00	>99.9
	14	$1.4 \times 10^3 \pm 1.7 \times 10^3$		
	28	$4.6 \times 10^5 \pm 4.9 \times 10^5$		

[a] apple pomace treatment, where control is no treatment. [b] Log reduction and % reduction of CFUs resulting from treatments relative to the control on that specific day.

Table S12: Average microbial counts on DRBC plates following treatment of whole apple pomace with 0.1–0.8 M acetic acid (AcOH), and change in microbial counts relative to the untreated control.

Treatment[a]	Day	Average microbial count ± std.dev (CFU/mL)	Log reduction[b]	% reduction[b]
Control	0	$3.9 \times 10^4 \pm 9.2 \times 10^3$		
	3	$2.5 \times 10^7 \pm 9.9 \times 10^6$		
	7	$1.1 \times 10^8 \pm 1.1 \times 10^7$		
pH 3.2, 0.10 M AcOH	0	$9.0 \times 10^3 \pm 7.1 \times 10^1$	0.63	76.8
	3	$3.6 \times 10^7 \pm 3.4 \times 10^6$	-0.15	-42.0
	7	$7.7 \times 10^7 \pm 2.3 \times 10^7$	0.15	29.2
pH 3.1, 0.20 M AcOH	0	$6.2 \times 10^3 \pm 1.1 \times 10^3$	0.80	84.0
	3	$3.0 \times 10^5 \pm 1.0 \times 10^4$	1.92	98.8
	7	$5.1 \times 10^7 \pm 6.4 \times 10^6$	0.33	53.2
pH 3.0, 0.40 M AcOH	0	$2.9 \times 10^3 \pm 4.9 \times 10^2$	1.13	92.6
	3	$3.0 \times 10^3 \pm 1.0 \times 10^2$	3.92	>99.9
	7	$2.2 \times 10^6 \pm 1.4 \times 10^4$	1.69	98.0
pH 2.8, 0.80 M AcOH	0	$1.7 \times 10^2 \pm 4.9 \times 10^1$	2.37	99.6
	3	$6.0 \times 10^1 \pm 1.4 \times 10^1$	5.62	>99.9
	7	$1.3 \times 10^2 \pm 2.8 \times 10^1$	5.93	>99.9
	14	$1.3 \times 10^3 \pm 1.6 \times 10^3$		
	28	$4.5 \times 10^5 \pm 4.7 \times 10^5$		

[a] apple pomace treatment, where control is no treatment. [b] Log reduction and % reduction of CFUs resulting from treatments relative to the control on that specific day.
